# A whole genome scan of SNP data suggests a lack of abundant hard selective sweeps in the genome of the broad host range plant pathogenic fungus *Sclerotinia sclerotiorum*

**DOI:** 10.1371/journal.pone.0214201

**Published:** 2019-03-28

**Authors:** Mark Charles Derbyshire, Matthew Denton-Giles, James K. Hane, Steven Chang, Mahsa Mousavi-Derazmahalleh, Sylvain Raffaele, Lone Buchwaldt, Lars G. Kamphuis

**Affiliations:** 1 Centre for Crop and Disease Management, Curtin University, Perth, Western Australia, Australia; 2 UWA School of Agriculture and Environment, The University of Western Australia, Perth, Western Australia, Australia; 3 Laboratoire des Interactions Plantes-Micro-organismes (LIPM), Université de Toulouse, INRA, Toulouse, France; 4 Agriculture and Agri-Food Canada, Saskatoon Research and Development Centre, Saskatchewan, Saskatoon, Canada; National Cheng Kung University, TAIWAN

## Abstract

The pathogenic fungus *Sclerotinia sclerotiorum* infects over 600 species of plant. It is present in numerous environments throughout the world and causes significant damage to many agricultural crops. Fragmentation and lack of gene flow between populations may lead to population sub-structure. Within discrete recombining populations, positive selection may lead to a ‘selective sweep’. This is characterised by an increase in frequency of a favourable allele leading to reduction in genotypic diversity in a localised genomic region due to the phenomenon of genetic hitchhiking. We aimed to assess whether isolates of *S*. *sclerotiorum* from around the world formed genotypic clusters associated with geographical origin and to determine whether signatures of population-specific positive selection could be detected. To do this, we sequenced the genomes of 25 isolates of *S*. *sclerotiorum* collected from four different continents–Australia, Africa (north and south), Europe and North America (Canada and the northen United States) and conducted SNP based analyses of population structure and selective sweeps. Among the 25 isolates, there was evidence for two major population clusters. One of these consisted of 11 isolates from Canada, the USA and France (population 1), and the other consisted of nine isolates from Australia and one from Morocco (population 2). The rest of the isolates were genotypic outliers. We found that there was evidence of outcrossing in these two populations based on linkage disequilibrium decay. However, only a single candidate selective sweep was observed, and it was present in population 2. This sweep was close to a Major Facilitator Superfamily transporter gene, and we speculate that this gene may have a role in nutrient uptake from the host. The low abundance of selective sweeps in the *S*. *sclerotiorum* genome contrasts the numerous examples in the genomes of other fungal pathogens. This may be a result of its slow rate of evolution and low effective recombination rate due to self-fertilisation and vegetative reproduction.

## Introduction

Spread of a favourable allele through a population due to positive selective pressure is known as a selective sweep [1,2]. When a favourable allele increases in frequency due to positive selective pressure, linked loci also increase in frequency. Linkage of non-selected loci decreases with distance from the favourable allele due to the effects of recombination [3]. Thus, immediately following a selective sweep, the genomic region surrounding the beneficial locus will be largely monomorphic throughout the population.

Several tests for selective sweeps have been developed based on this understanding [4–6]. Such tests have been applied to numerous organisms to study the footprints of positive selection in genomes [7]. In most studies, evidence of selective sweeps localised to particular populations has been observed. For example, 346 genomic regions in *Drosophila melanogaster* were found to exhibit a significantly high degree of fixation between populations from Europe and Africa. Genes within these regions were enriched for various functional activities including transcriptional regulation [8]. In humans, numerous population specific signals of positive selection have been identified and linked with genes involved in processes such as skin pigmentation, immunity, heat shock and olfactory perception [9–12].

Plant pathogenic fungi may be useful organisms for the study of positive selection. This is because they likely undergo rapid adaptation to anthropogenic selective pressures such as dissemination to naive environments and introduction of new crop varieties or antifungal chemistries [13–20]. Selective sweep scans of several plant pathogenic fungi have been published recently. These include analyses of the wheat pathogen *Zymoseptoria tritici*, the carnation pathogen genus *Microbotryum* and the barley pathogen *Rhyncosporium commune*. In *Z*. *tritici*, selective sweeps were found across 0.5–4% of the genome, in four field populations. Swept genome regions in this pathogen contained secondary metabolite clusters, transporters and genes possibly involved in host colonisation [21]. In *Microbotryum*, selective sweeps covered 1% and 17% of the genomes of the sister species *M*. *lychnidis-dioicae* and *M*. *silenes-dioicae*, respectively. Several candidate genes that may have roles in infection were identified in these species, including a cysteine-rich CEFM domain protein, oligopeptide transporters and major facilitator superfamily tranporters [22]. In *Rhynchosporium commune*, 39 genomic regions with signatures of positive selection were detected across the 27 genome scaffolds [23]. Among genes associated with these selective sweeps were a multiantimicrobial extrusion multidrug transporter and enzymes involved in degradation of plant cell walls.

To gain further insight into positive selective pressure in plant pathogens, we examined the genome of the fungus *Sclerotinia sclerotiorum* for evidence of selective sweeps. The genome sequence of *S*. *sclerotiorum* was recently sequenced to completion using PacBio technology and has been annotated using extensive RNA sequencing data and manual curation, which makes it an attractive resource for genome scale analyses of selection [24].

*S*. *sclerotiorum* is a host generalist that infects more than 600 species of plant [25]. It has been globally disseminated and is present in numerous environments where it causes significant yield losses in economically important crops [26]. Several studies have demonstrated genotypic differentiation of *S*. *sclerotiorum* populations from different geographic regions. For example, Kamvar et al. showed that populations of *S*. *sclerotiorum* from Mexico shared no multilocus haplotypes with populations from the United States, Australia and France [27]. Similarly, Lehner et al. showed that 29% of variance within a sample of isolates from southeastern Brazil and the United States was explained by differences between populations from the two regions [28]. And, echoing these findings, Clarkson et al. found that populations of *S*. *sclerotiorum* from the UK, Norway and Australia shared no multilocus haplotypes [29].

To date, selective sweep scans in plant pathogenic fungi have focussed on narrow host range species that exhibit prolific sporulation and rapid evolution in response to host resistance genes and fungicides. In contrast to these pathogens, the propensity for clonality, broad host range and monocyclic nature of *S*. *sclerotiorum* may lead to a slower rate of evolution. This theoretically slower rate of evolution is supported empirically by the observation that *S*. *sclerotiorum* has, so far, only developed resistance to a single class of fungicides, the MBCs. Furthermore, the time to resistance development (FDR) in *S*. *sclerotiorum* was 25 years–slower than any other pathogen for the same group of fungicides. This is 23 years longer than the FDR for the fast evolving pathogens *Didymella bryoniae* and *Penicillium expansum*. For a different class of fungicides, the QoIs, fungicide resistance evolved in a mere 7 years for *Z*. *tritici*, which was the subject of one of the previously mentioned selective sweep studies [30]. Thus, the questions remain, does a likely slowly evolving pathogen exhibit evidence of recent and strong selective sweeps? If so, how prevalent are they and what kinds of genes do they affect?

To identify which genomic regions may have undergone strong positive selective pressure in different populations of *S*. *sclerotiorum*, we sequenced the genomes of a collection of 25 isolates from Canada, France, the USA, South Africa, Morocco and Australia. These isolates appeared to be sampled from two major populations, which were composed of isolates from Europe and North America and isolates from Australia and Morocco. A single candidate selective sweep was identified in one of these populations, covering less than 0.001% of the genome. There was a single gene within 10 Kb of this selective sweep, which encoded a major facilitator superfamily transporter that was only negligibly expressed at late stages of *Brassica napus* infection. We speculate that a slow rate of evolution and extensive self-fertilisation in *S*. *sclerotiorum* may have led to the striking absence of strong selective sweeps.

## Methods

### Fungal isolates

In this study, the reference isolate 1980 (NCBI Bioproject ID: PRNJA348385) was used as a reference for genotype calling in most analyses, and as a result is not explicitly included as a sample. For the whole genome alignment described, the reference isolate was explicitly included since this analysis requires no designated reference strain. Two originally selected isolates from a panel of 27 re-sequenced strains were dropped as they were likely not *S*. *sclerotiorum*.

Thus, a total of 26 isolates were analysed, including the reference isolate, for which a continuous chromosome sequence is available (NCBI Bioproject ID: PRJNA348385) [24]. These also included isolates from Australia (x 12), Europe (x 5), North America (x 6) and Africa (x 2). The names of the isolates, host of origin (if known), location, year collected, accession numbers (where applicable) and names of isolate stock collections (where applicable) are summarised in S1 Table. The isolates originate from regions with a range of different climates, which include the following distinct climate types according to the Köppen climate classification scheme [31]: Csa, hot summer Mediterranean; Csb, warm summer Mediterranean; Cfb, Oceanic;Dfb, humid continental.

### DNA extraction and sequencing of fungal isolates

DNA from the isolates S55, Ss44, Ss45, Sssaf, SsChi, BloC104, BloC014, P134, P163, FrB5, SK35, 321, MB52, MB21 and AB29 was extracted and sequenced in a previous publication [25]. DNA from all Australian isolates apart from CULm and CULa was extracted and sequenced using the following method: sterile sclerotia of each isolate were plated onto full strength potato dextrose agar (PDA) and grown for 4–10 days at 20°C in the dark. Mycelium from the edge of each colony was harvested and DNA was extracted using a modified version of the alkaline lysis method described by Rollins [32]. In brief, following alkaline lysis, DNA solutions were partitioned using 24:1 chloroform:isoamyl alcohol and precipitated with isopropanol and lithium chloride before being resuspended in 30 μl of milliQ water + 1 μl of RNAse A (10 mg/ml). High quality DNA was sequenced on the Illumina Hiseq platform using 2 x 250 bp paired end sequencing.

DNA from the isolates CULm and CULa was extracted and sequenced using the following method: A seven day old fungal culture grown on PDA was harvested and homogenised with pestle and mortar by adding 1 mL of half strength potato dextrose broth (PDB). The suspension was transferred to a 250 ml flask containing 100 ml of half strength PDB. The flask was shaken at 100 rpm and kept in darkness for 4 days at 20°C. After 96 hours post inoculation, the fungal culture was filtered through sterile cheese cloth and washed twice with sterile distilled H_2_O to remove any excess nutrients. The culture was freeze-dried overnight before genomic DNA extraction using the CTAB method [33]. DNA concentration was quantified using a Qubit 2.0 fluorometer (Life Technologies) and assessed by gel electrophoresis for quality control. Sequencing was performed by the Australian Genome Research Facility using the Illumina Hiseq platform with 2 x 250 bp paired end sequencing.

All genomic DNA sequencing reads generated for this study have been deposited to the GenBank Sequence Read Archive under the bioproject numbers PRJNA449247 (for isolates CULm and CULa) and PRJNA516948 (for all other isolates including non-*S*. *sclerotiorum* samples).

### *HSP60* phylogeny to assign isolates to the species *Sclerotinia sclerotiorum*

To assess whether isolates were the species *S*. *sclerotiorum*, a phylogenetic tree was built using previously published heat shock protein 60 (*HSP60*) gene sequences [34] in conjunction with *HSP60* gene sequences derived from *de novo* assemblies. GenBank accessions for previously published *HSP60* sequences are in S2 Table.

*HSP60* sequences were extracted from *de novo* assembled contigs (*de novo* assembly of fungal isolate genomes is detailed in a later section ‘Quality filtering and *de novo* assembly of fungal isolate genomes’). Exonerate version 2.2.0 [35] was used to align the previously published *HSP60* gene sequence for *S*. *sclerotiorum* to each *de novo* assembly. The commands ‘—bestn 1—mode est2genome’ were used to identify the single best alignment for each genome. The coordinates of these alignments were extracted to produce browser extensible data (BED) formatted files containing strand information. Bedtools version 2.25.0 [36] was then used with the commands ‘getfasta -s’ in conjunction with these files and the *de novo* assemblies to extract regions aligning to the published *HSP60* sequence. The option ‘-s’ was used to take into account strand information of the alignments.

The isolate specific and previously published *HSP60* sequences were aligned using ClustalW version 2.1 [37] with default settings. The appropriate nucleotide substitution model was chosen using JModelTest version 2.1.10 (v20160303) [38] with the settings ‘-s 11 -f -i -g 4 -AIC -BIC -AICc -DT -p -a’. The Akaike Information Criterion (AIC) test was used to determine which nucleotide substitution model to use. The model used was the ‘transitional model’ (TIM) first described in [38].

An *HSP60* based phylogeny was constructed from the alignments using PhyML version 20120412 [39] with the commands ‘-b 100 -d nt -n 1 -m 012230 -f 0.25,0.25,0.25,0.25 -c 4 -a e—no_memory_check—r_seed 12345 -o tlr -s BEST’ (with the TIM substitution model). Support for branches was assessed using 100 bootstrap tests. The species *Botrytis cinerea* and *Monilinia fructigena* were used as outgroups.

### Quality filtering and *de novo* assembly of fungal isolate genomes

Paired reads from each of the *S*. *sclerotiorum* isolates were assessed for adapter contamination and poor quality sequence content using FASTQC version 0.11.4 [40]. Illumina data that required adapter and poor quality sequence removal were filtered using Trimmomatic version 0.36 [41]; the adapter file used with Trimmomatic contained sequences for the TruSeq LT and v1/v2 kits. Information on whether isolates were quality and adapter filtered is included in S1 Table. The paired Illumina reads from each isolate were *de novo* assembled using the A5-miseq pipeline version 20160825 [42] with default settings.

### Mapping of Illumina reads to the reference genome

Paired reads for each of the isolates were mapped to the reference genome using Stampy version 1.0.31 with default settings [43]. Both paired reads and singletons that survived the previously described quality filtering step (see ‘Quality filtering and *de novo* assembly of fungal isolate genomes’) were mapped to the reference genome. Reads were first mapped using BWA MEM version 0.7.5a-r405 [44] and resulting binary alignment map (BAM) files were used as input to Stampy.

### Variant calling and quality filtering

Following alignment, the Genome Analysis Toolkit (GATK) version 3.7-0-gcfedb67 [45] was used to call variants between Illumina reads and the reference. The module ‘HaplotypeCaller’ with the setting ‘-ploidy 1’ was used to call initial variants between reads and the reference assembly. Then, the module ‘SelectVariants’ was used to select only SNPs. The module ‘VariantFiltration’ was then used to quality filter these SNPs based on the values ‘QD < 2.0’, ‘AF < 1.0’, ‘FS > 60.0’, ‘MQ < 40.0’, ‘MQRankSum < -12.5’ and ‘ReadPosRankSum < -8.0’; the filter flag ‘snp_filter’ was applied to the resulting variant call format (VCF) file.

Following this, the latter two procedures were followed for InDel polymorphisms derived from the initial HaplotypeCaller step. For the ‘SelectVariants’ step considering InDels, the filtering parameters ‘QD < 2.0’, ‘AF < 1.0’, ‘FS > 200.0’, ‘ReadPosRankSum < -20.0’ were used. The filter tag ‘indel_filter’ was applied to the resulting VCF file. The two VCF files and the original VCF file produced by HaplotypeCaller were then merged using the ‘CombineVariants’ module. The settings for this module were ‘-genotypeMergeOptions PRIORITIZE -priority a,b’.

The modules ‘BaseRecalibrator’ and ‘PrintReads’ were then used in conjunction with the merged VCF to recalibrate the BAM alignment. The merged VCF contained higher confidence SNPs and InDels that had passed the quality filtering steps specified in the previous paragraph. HaplotypeCaller was subsequently used, with the settings ‘-ploidy 1’ and ‘—emitRefConfidence GVCF’ to generate a genomic VCF (GVCF) file using the recalibrated BAM.

The GVCF files produced using the recalibrated BAM file were merged using the GATK module ‘GenotypeGVCFs’. Two merged VCFs were created based on two groups of individuals, isolates from Europe and North America, and isolates from Australia and Morocco. A further merged VCF containing all isolates was also created for analysis of population structure. The joint genotyped VCFs were then hard-filtered again using the GATK module ‘VariantFiltration’. Genotypes with a depth of less than 10 or more than 150 and a GQ score of < 40 were removed. SNPs and InDels that passed this filtering step were retained with the GATK module ‘SelectVariants’. The maximum depth filter of 150 was used to remove repeat-induced alignments and was chosen because it was at the upper-end of the bell curve for coverage in all isolates and below the long tail of repeat-induced coverage (Fig A in S1 File).

### Construction of a whole genome phylogenetic network

The *de novo* assembled genomes produced using the A5 pipeline were used to construct a SNP based distance matrix using Andi version 0.9.6.1 [46]. From the distance matrix produced by Andi, a Neighbor-net network [47] was constructed using splitstree version 4 [48]. A K-mer based distance matrix was also generated using Mash version 2.0 [49]. This distance matrix was also used to build a Neighbor-net network using splitstree.

### Principal component and population structure analysis of genotypic variation and analysis of linkage disequilibrium decay

The filtered VCF file containing information from all isolates produced in the previous section ‘Variant calling and quality filtering’ was subjected to a principal component analysis (PCA) using the R Bioconductor package ‘SNPRelate’ version 1.4.2. First, SNPs in strong linkage disequilibrium were filtered using PLINK version 1.90b4.1 [50]. To do this, the VCF file was converted into a PED file using VCFTools version 0.1.15 [51]. This file was then filtered with PLINK using the settings ‘—indep-pairwise 50 5 0.5’. The coordinates of the resulting SNPs were extracted from the PED file using Awk and then passed to VCFTools to use as a filter with the ‘—positions’ argument. Then, in R, the function ‘snpgdsPCA’ was used with the setting ‘autosome.only = FALSE’ to perform the PCA. Eigenvectors one and two were plotted on the x and y axes, respectively.

Population structure was further assessed using ADMIXTURE version 1.3.0 [52]. For this analysis, the PLINK file that had been filtered for SNPs showing strong linkage disequilibrium was used. ADMIXTURE was run for K = 1..10 with the option ‘—cv = 10’ for cross-validation.

To calculate linkage disequilibrium decay, a random 30% of the SNPs for each group were selected using the GATK module ‘SelectVariants’ with the settings ‘-fraction 0.3’, ‘—excludeFiltered’, and ‘—nonDeterministicRandomSeed’. Pairwise linkage disequilibrium was calculated between all SNPs between 1,000 and 999,999 bp apart using PLINK with the settings ‘—allow-extra-chr’, ‘—r2’, ‘—ld-window-r2 0’, ‘—ld-window-kb 1000’, ‘—ld-window 999,999’. The mean value of linkage disequilibrium across 10 Kb end-to-end sliding windows from any given SNP was then calculated. Loess curves were fit to the mean linkage disequilibrium decay values for the sliding windows with the R function ‘loess’. To determine the approximate distance required to reach 50% linkage disequilibrium decay, the last window start before the mid point between highest and lowest LD, going from high to low LD.

### Determination of polymorphism effects on coding sequences

The software package SNPEff version 4.3i [53] was used with default settings to determine what coding sequences were affected by SNPs in the filtered VCF from the previous section ‘Variant calling and quality filtering’. The GFF3 file containing reference coding sequences considered in this analysis was downloaded from NCBI (Bioproject number: PRJNA348385). The SNP classifications obtained from this analysis were ‘LOW’, ‘MODERATE’ and ‘HIGH’. These correspond to synonymous mutations with a theoretically lower effect on coding sequences, non-synonymous mutations that may change amino acid sequence without causing major disruption of protein function and highly disruptive non-synonymous mutations that cause alterations such as frame shifts or premature stop codons.

To identify possible sites of transposon insertions into genes, RetroSeq version 1.41 [54] was used with default settings. Read mappings in BAM format from the previous section ‘Mapping of Illumina reads to the reference genome’ were supplied to RetroSeq with the Repet repeat annotation previously generated by Derbyshire et al. [24].

### Estimation of recombination rate in the *Sclerotinia sclerotiorum* populations

Recombination rate was estimated for both populations using LDhat [55]. The two VCF files for the populations were converted to LDhat unphased genotype files. A finite sites estimate of Watterson’s theta per site was calculated for each chromosome. Lookup tables were then created using the LDhat module ‘complete’ with the settings ‘-rhomax 100 -n_pts 101’. The LDhat module ‘interval’ was then used with the settings ‘-its 5000000 -bpen 10 -samp 5000’. The LDhat module ‘stat’ was then run with the setting ‘-burn 100’. This produced recombination rates for each chromosome. The recombination rate used in the MS simulations was the average of the two average chromosome recombination rates for the two populations.

### Modelling of demographic history of *Sclerotinia sclerotiorum* samples

Dadi version 1.7.0 [56] was used to model demographic history of two *S*. *sclerotiorum* samples representing two genotypic groups referred to as ‘population 1’ and ‘population 2’ henceforth. These groups were decided on based on the ADMIXTURE, PCA and neighbor-net trees described in a previous section. Population 1 consisted of 11 isolates from Europe and North America and population 2 consisted of nine isolates from Australia and one isolate from Morocco.

Before conducting the analysis, the VCF file containing genotype data for both populations was filtered to remove SNPs with effects on coding sequences (identified using SNPEff) using negative regex expression searches with ‘grep’. The Python script ‘easySFS.py’ (available at the time of writing at https://github.com/isaacovercast/easySFS) was then used to convert the VCF into a site frequency spectrum (SFS) formatted file, as specified by Dadi. Several different models were fit to the joint SFS, including a standard neutral model, which assumed no demographic events and a random sampling of two groups of individuals from the same population; a single population size change followed by exponential growth and random sampling of the one population; a two step model with an ancient population size change, followed by exponential growth, then a split and further exponential growth, with no migration between populations; a two step model with the same events that also included equal migration between populations; a single step model involving a population split and instantaneous size change and no growth, with an equal migration rate; a two step model with an ancient population size change, followed by a population isolation model where the two populations are made up of fractions of the ancestral population that grow exponentially with unequal migration; and, finally, a one step model similar to this but with no ancestral population size change (see results for an illustration of these models and their fits to the data).

Parameters were optimised using the ‘optimize_log’ function. The optimised parameters were then used as starting parameters and randomised 20 times using the ‘perturb_params’ function. The randomised parameters were then re-optimised using the ‘optimize_log’ function. Results of the 20 optimisations based on the perturbed parameters were assessed to determine convergence of parameter sets based on SFS data. The model with the best fit to the data was used to specify 10,000 simulations in MS for the selective sweep testing [57]. Theta was derived from the optimised model constructed using Dadi and recombination rate was derived from LDhat estimates. With each simulation, the same number of samples as were in each population were produced for downstream analyses.

Model fit was determined based on comparison of log likelihoods. In instances where there was not a large and obvious change in log likelihood, the improvement in fit of one model over another was assessed using coalescent simulations. 10,000 MS simulations were run using the model with the lower log likelihood, again using recombination rate estimated by LDhat, and the improvement in model fit to the simulation from the model with the lower log likelihood to the model with the higher log likelihood was recorded. The distribution of likelihood improvements was assessed to determine whether additional model parameters were fitting noise or detecting additional signal in the data. This simulation-based approach is favourable over the traditional AIC or BIC approaches for model selection as it accounts for recombination and does not bias the estimate towards rejection of the less parameter-dense model.

### Detection of selective sweeps

Both the real data in the population 1 and population 2 VCF files and the simulated SFS data were scanned for selective sweeps using SweeD version 3.3.2 [5]. For each chromosome, the analysis was run separately on a grid size corresponding to positions approximately 5 Kb apart. These analyses were run on all SNPs in the filtered VCF files produced in the previous section ‘Variant calling and quality filtering’. For the simulated SFS data, SweeD was run for the same number of chromosomes as there were samples for each population (population 1 = 11, population 2 = 10). Simulated chromosomes were scanned on a grid of 800 points and were 4 Mb long. Thus, the MS data were also analysed at approximately 5 Kb intervals. A total of 10,000 MS simulations were analysed and the maximum composite likelihood ratio (CLR) value for all combined simulations was used as a threshold for detection of selective sweeps. To find genes associated with these regions, Awk was used to generate a BED formatted file containing coordinates 1 / alpha either side of each putative selective sweep. The calculation 1 / alpha is used to obtain the extent of a selective sweep in Nielsen et al. (2005), where alpha is the strength of positive selection. The Bedtools module ‘intersect’ was then used to determine which genes were within these regions.

For each population, Tajima’s D was calculated across a 5 Kb sliding window using the R package ‘PopGenome’ version 2.2.4. Fixation index (F_ST_) between populations was also calculated across a 5 Kb sliding window using this package.

### Analysis of genes overlapping selective sweeps

Candidate genes underlying selective sweeps were identified using the coordinates of the selective sweeps + and– 5 Kb either side, and information on SNP impacts on coding sequences and transposon insertions.

## Results

### Assignment of isolates to the species *Sclerotinia sclerotiorum*

To confirm that fungal isolates belonged to the species *S*. *sclerotiorum*, a phylogenetic tree was built using previously published *HSP60* gene sequences in conjunction with sequences from *de novo* assemblies. Based on inference from this tree, two originally selected isolates ‘Ss45’ and ‘SsChi’ were dropped from further analyses as they appeared to lie outside the *S*. *sclerotiorum* clade. Ss45 was not in any known *Sclerotinia* spp. clades whereas SsChi appeared to be in a clade with known *S*. *minor* sequences (Fig B in S1 File).

### Population substructure is associated with geography

**Fig 1:**
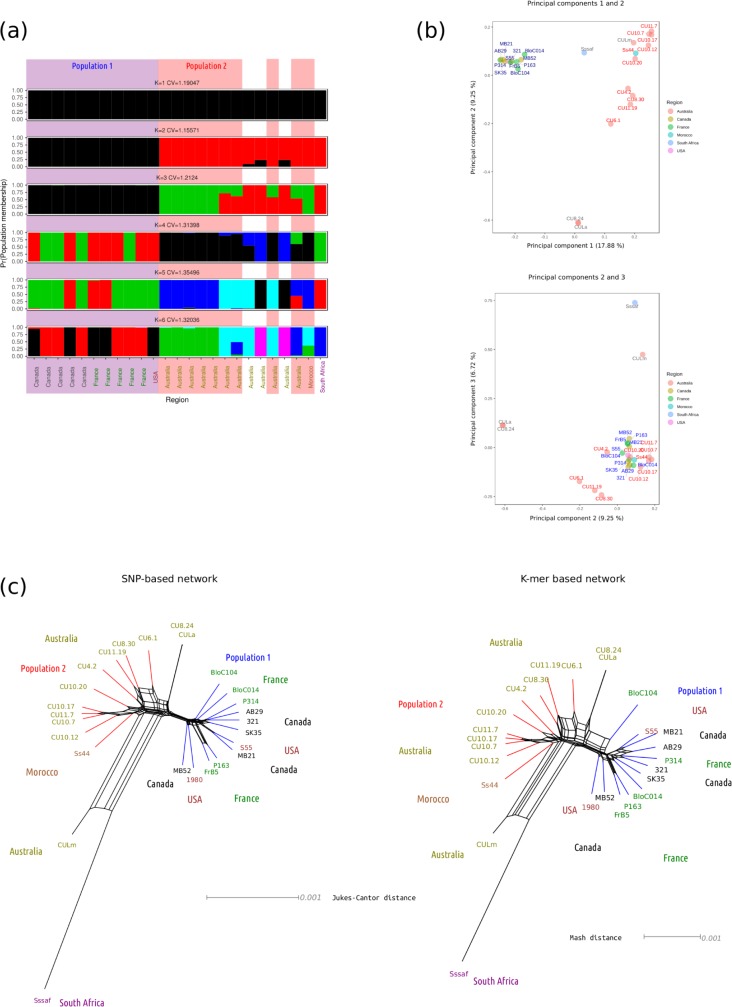
Analysis of population structure in a panel of 25 global isolates of *Sclerotinia sclerotiorum*. (a) Population structures identified with K = 1..6 predefined populations. The y axis represents probability of population membership, the x axis represents different isolates (x axis labels are coloured according to isolate region of origin) and different colours represent the K = 1..6 populations. The number of populations with the lowest cross validation error was K = 2 (CV = 1.155). The two populations used throughout the selective sweep scans, population 1 (North American and European isolates) and population 2 (Australian isolates and a single Moroccan isolate) are blocked in blue and red shading, respectively. (b) Principal component analysis of population structure. Top panel: principal component 1 (17.88% of variance) is plotted on the x axis and principal component 2 (9.25% of variance) is plotted on the y axis. Points are coloured according to isolate region of origin (defined in the legend to the right) and labelled with isolate names in blue for population 1 membership and red for population 2 membership. The grey labels indicate that isolates were not included in further analyses as their genotypes were outliers with respect to the rest of the sample. Bottom panel: the same as for the top panel but for principal components 2 (9.25% of variance) and 3 (6.72% of variance). (c) Neighbor-net networks showing relatedness of isolates. Left panel: a SNP-based network derived from a genetic distance matrix produced using a whole genome alignment. Tip labels are coloured and labelled according to region of origin and branches are coloured according to whether the isolate is in population 1 (blue) or population 2 (red). The scale bar represents the Jukes-Cantor genetic distance. Right panel: the same as for the left panel but using a K-mer based approach. The scale bar represents Mash distance. Note that in both the left and right panel, the reference isolate ‘1980’, which was isolated from Soybean in the United States, is included. This is because its sequence was not implicit in the whole genome alignment as it was in the genotypic data used for Fig 1A and 1B.

Whole genome neighbor-net networks, and ADMIXTURE and principal component analyses were used to test for population substructure among the 25 isolates and the reference strain. The ADMIXTURE analysis showed that the value of K (number of hypothetical ancestral populations) with the lowest cross validation error was two (cross validation error = 1.15571). This grouped isolates into two clusters, consisting of those from North America and Europe and those from Australia and Africa. However, cross validation error did not increase much with K, and K values of three, four, five and six (cross validation error = 1.2124, 1.31398, 1.35496 and 1.32306, respectively) suggested further population structure within the Australian and African population. For K values of three, four and five there was a consistent grouping of Ss44 from Morocco and five Australian isolates into a single population cluster. For all values of K tested, there was little overlap in ancestral population membership between isolates from North America and Europe and isolates from Africa and Australia (Fig 1).

The ADMIXTURE analysis was supported by a PCA, which showed that principal component 1, which explained 17.9% of variance, split the isolates into two groups, isolates from North America and isolates from Australia and Africa. The second principal component explained 9.25% of the variance and was mainly associated with differences between two isolates from Australia, CU8.24 and CULa, and the rest of the Australian and African isolates. Examination of principal component three further separated individuals in the Australian and African cluster from the isolate Sssaf, from South Africa, and CULm from Australia (Fig 1).

The two neighbor-net phylogenetic networks constructed from SNPs and K-mers showed a similar clustering of isolates, and also suggested that Sssaf, CULm, CULa and CU8.24 were outliers. All European and North American isolates formed a single cluster in the network and nine Australian isolates and Ss44 from Morocco grouped together among the rest of the isolates (Fig 1). Based on these results, we defined two populations for the selective sweep analyses. These were population 1, which consisted of isolates from North America and Europe, and population 2, which contained Ss44 from Morocco and nine of the Australian isolates (Fig 1).

**Fig 2:**
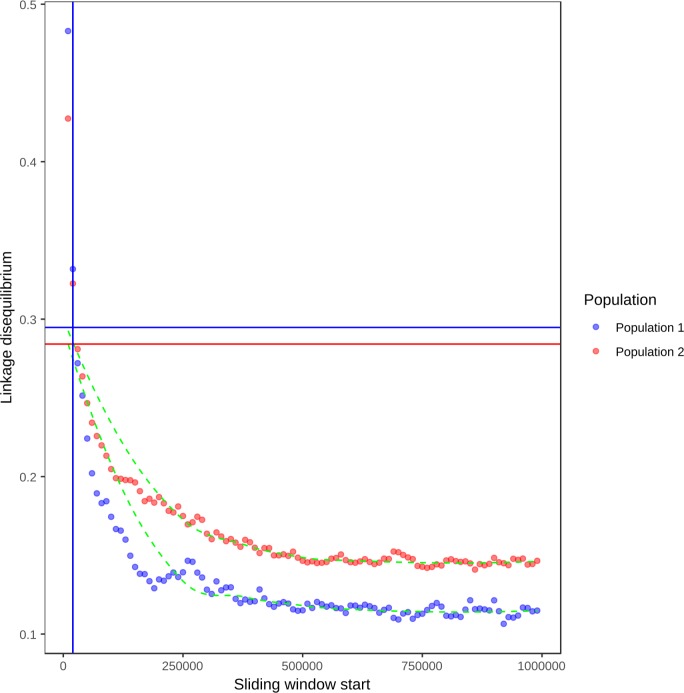
Linkage disequilibrium decay in the two defined *Sclerotinia sclerotiorum* populations. Mean linkage disequilibrium (x axis) is plotted for 10 Kb sliding windows up to 1000 Kb from each SNP. The green dashed lines are LOESS curves fit to the mean linkage disequilibrium values. Points are in blue for population 1 (North American and European isolates) and red for population 2 (Australian isolates and a single Moroccan isolate). The vertical lines indicate the position at which linkage disequilibrium decayed to half its maximum value in each population, with population 1 in blue and population 2 in red (red and blue fall together at the same window start, so the red line is not visible). The horizontal lines represent half the maximum linkage disequilibrium for each population, with population 1 in blue and population 2 in red.

To test whether the two populations exhibited evidence of recombination, we estimated recombination rate using LDhat and also calculated linkage disequilibrium (R^2^) in 10 Kb sliding windows from each SNP in a randomised set of 30% of the total SNPs. LDhat estimates suggested recombination rates of 0.000172 and 0.0000123 for population 1 and population 2, respectively (S3 Table). For both populations, LD decayed to 50% its maximum value at approximately 20 Kb. For population one, maximum LD was 0.48 and for population 2, it was 0.43. Minimum LD for population 1 was 0.11 and for population 2, it was 0.14. This showed that both populations exhibited evidence of linkage disequilibrium decay with distance (Fig 2). This echos the findings of Attanayake et al. (60) who inferred outcrossing in populations of *S*. *sclerotiorum* based on linkage disequilibrium decay. Based on these observations of likely sexual recombination, we believe that both the selective sweep and demography analyses are applicable to this data set.

### Demographic modelling suggests an ancient split with a large migration rate between the two populations

**Fig 3:**
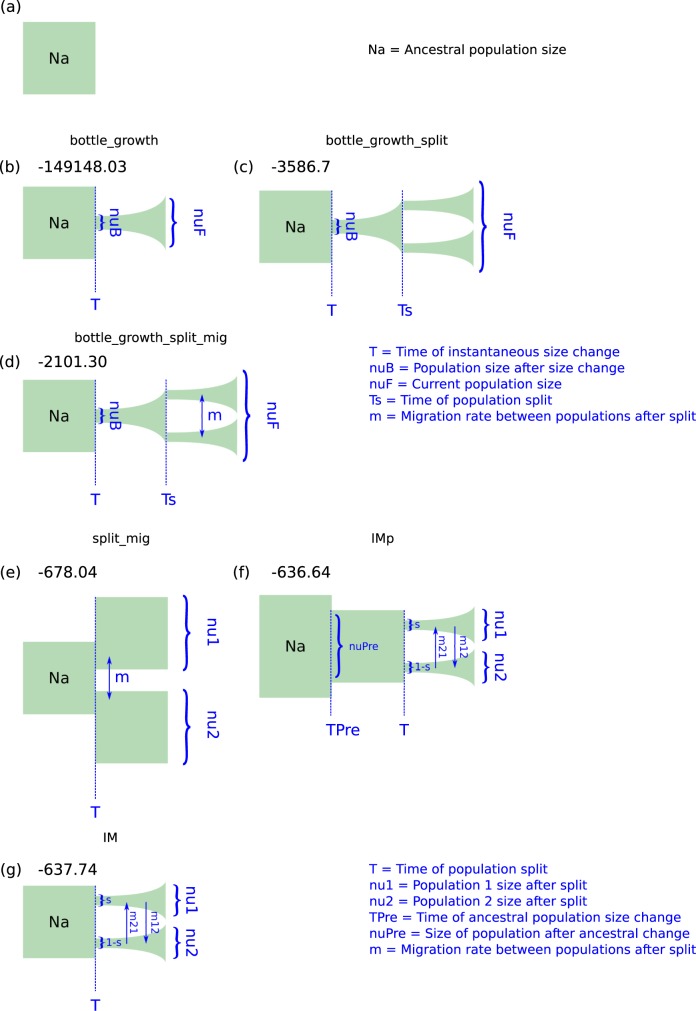
Demographic models fit to the joint site frequency spectrum (SFS) for the two defined *Sclerotinia sclerotiorum* populations. (a) The theoretical ancestral population size (Na) is depicted as a green block, which forms the end point for model coalescence. (b) A single population model fit to the sfs, considering both populations as a single population. This model takes the parameters nuB, which refers to a population size change at time T, followed by exponential growth to reach a current population size of nuF; it’s log likelihood was -149148.03. (c) A two population model fit to the joint sfs for the two populations. This model takes the parameters nuB, which refers to a population size change at time T, followed by exponential growth until time Ts. At time Ts, the population splits into two, and both populations then exponentially grow to reach a current population size of nuF; it’s log likelihood was -3586.7. (d) The same as for (c) but with an additional parameter m, which allows for migration between the populations after the split; it’s log likelihood was -2101.30. (e) A simple model defining a population split at time T, with an instantaneous change in population sizes with migration (m) between the two populations. At the time of sampling, the populations have sizes nu1 and nu2; it’s log likelihood was -678.04. (f) Similar to (e) but with an ancestral population size change at time TPre to size nuPre before the split at time T. The split results in fragmentation of the ancestral population with a proportion s going into one population and a proportion 1-s going into another. Following the split, the two populations undergo exponential growth to reach sizes nu1 and nu2, with unequal migration allowed between the populations with the parameters m21 and m12. This model’s log likelihood was -636.64. (g) The same as for (f) butwithout the ancestral size change in the population. The three models (e-g) were obviously better fits for the data than (b-d). Coalescent simulations suggested that there was no improvement in model fit over (e) with the additional parameters in (f) and (g).

The Python module Dadi was used to identify recent demographic events in the two populations of isolates. The standard neutral model, used as a baseline for model testing, did not fit the data well, with a log likelihood of -149878.21. This was the worst model fit of all tested models. The three models that fit the data relatively plausibly were a population split with instantaneous size change and equal migration between populations (Split Migration ‘split_mig’), an ancestral population size change followed by population fractionation and exponential growth with unequal migration (Isolation Migration pre ‘IMp’) and a population fractionation followed by exponential growth with unequal migration (Isolation Migration ‘IM’). These models had log likelihoods, given the data, of -678.04, -636.64 and -637.64, respectively. The next best fitting model had a log likelihood of -2101.30. This model was also more parameter-dense than the split_mig model, suggesting it was not a good fit for the data (Fig 3).

**Fig 4:**
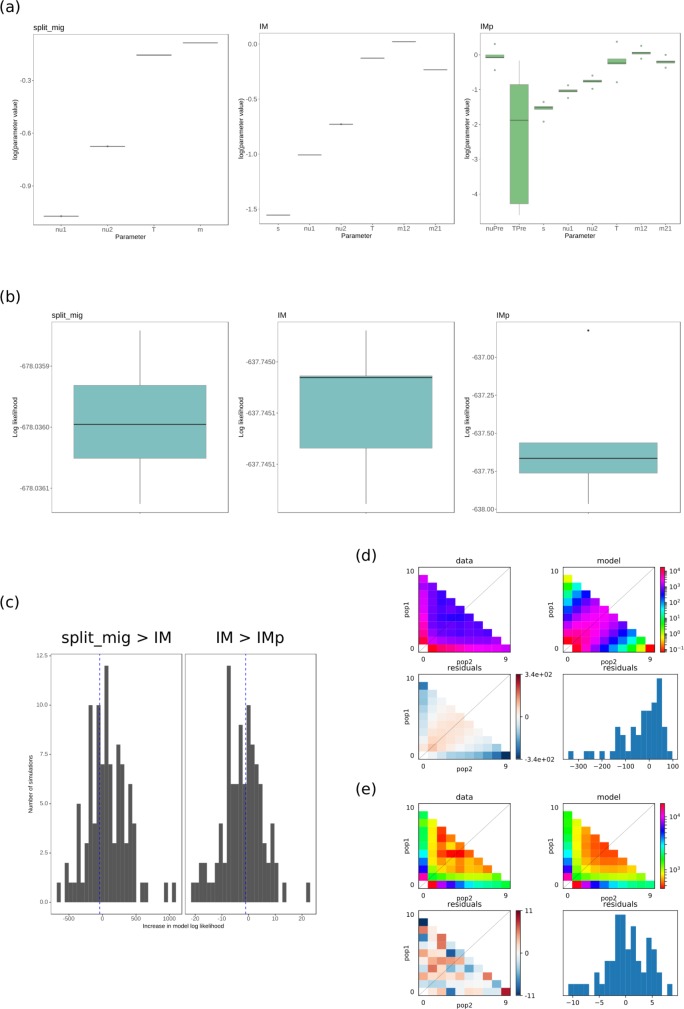
Model fits to sfs data from the *Sclerotinia sclerotiorum* populations. (a) Results of parameter optimisations for the three best fitting models, split migration (“split_mig”, corresponding to Fig 3E, log likelihood = -678.04), isolation migration (“IM”, corresponding to Fig 3G, log likelihood = -637.74), isolation migration with an ancestral population size change (“IMp”, corresponding to Fig 3F, log likelihood = -636.64). Model parameters are indicated on the x axis and the interquartile range of the logarithms of optimised parameters for five out of 20 optimisations (from random starting parameters) with the highest log likelihoods are shown on the y axis as boxes and whiskers. The black horizontal bars represent median values. Most parameters converged for most models at higher log likelihoods. Parameters were least convergent for the IMp model, especially for the estimation of ancestral population size change. (b) Interquartile range of model log likelihoods plotted as boxes and whiskers on the y axis for the 5 best fitting models after randomisation and re-optimisation of parameters 20 times. Black horizontal bars represent median values. (c) Results of 10,000 coalescent simulations with the best fitting models. Left panel: with each simulation, the split_mig coalescent frequency spectrum was fit to the split_mig diffusion approximated frequency spectrum and the IM diffusion approximated frequency spectrum. The difference between these two log likelihoods was calculated. The bars represent distribution of differences in log likelihoods (x axis) for each randomisation and the blue vertical dashed line represents the actual difference in log likelihood for the split_mig model and IM model. Since this line falls within the middle of the distribution, the IM model was likely fitting noise rather than additional signal in the data. Right panel: the same as for the left panel but testing the improvement in fit of the IM to IMp models. Again, improvement in log likelihood of IMp over IM was likely due to fitting of noise rather than additional signal in the data. (d) Residual plot of standard neutral model compared with the *Sclerotinia sclerotiorum* two population joint frequency spectrum. Upper panel: on the x axis is minor allele count for population 1 (pop1) and on the y axis is minor allele count for population 2 (pop2). Colouring represents the scaled frequency of the two population allele counts. The data are on the left and the standard neutral model is on the right. Lower panel: Poisson residual deviation of observed frequency spectra from model frequency spectra. The left is the same as the plots shown in the upper panels but instead of the frequency spectrum, it shows poisson residuals for each point in the frequency spectrum. The right is a histogram of residuals, with residual value on the x axis and frequency on the y axis. (e) The same as for (d) but for the split_mig model. This model was chosen as it was clearly a better fit than the standard neutral model and models with more parameters, i.e. IM and IMp, had only marginally improved fit by fitting noise (as evaluated in (c)).

For the three best fitting models, parameter values were generally convergent for the 5 optimisation runs with the highest log likelihoods (Fig 4). Parameters were the least convergent for the IMp model, especially the parameter estimating the time of ancestral population size change (TPre).

To determine whether the more parameter-dense models IM and IMp fit the data better than the split_mig model or were just fitting noise, 10,000 MS simulations were run for the split_mig and IM models with the recombination rates estimated from LDhat. The differences in model fit between either split_mig and IM or IM and IMp to the split_mig and IM simulations, respectively, were calculated for each randomisation and the difference in fit to the actual data for these models was compared with the distributions. This showed that there was no significant increase in model fit from split_mig to the IM or from the IM to IMp models (Fig 4). Therefore, we chose the less parameter-dense split_mig model for MS simulations used to define a threshold for the selective sweep scan. Fig 4 shows the fit of the standard neutral model to the data set and the split_mig model to the data set.

The optimised parameters for each model are given in S4 Table. For the split_mig model, optimised parameters were nu1 = 0.34, nu2 = 0.5, T = 0.85, m = 0.9. The parameters nu1 and nu2 describe the sizes of population 1 and population 2 after the split, as a proportion of the ancestral population size. We would infer from this that population 2, the Australian and African population, was larger than population 1, the North American and European population, after they diverged from their ancestral population. The parameter T describes the time, in 2Ne, from the present that the split occurred. A T of 0.85, given the optimal model theta of 32507.98 and a fungal mutation rate of 1.9*10^−10^ (estimated from yeast [58]), would place the divergence time in the range of 1–16 million years before present (assuming an (unknown) generation time, estimated based on sclerotial survival, of 1–8 years). The symmetric migration rate between populations estimated by this model, the parameter m, was 0.92. This parameter is in units of ancestral population size (4Ne) and suggests a moderate to low level of historical gene flow between the populations.

### A single candidate selective sweep is present in population 2

**Fig 5:**
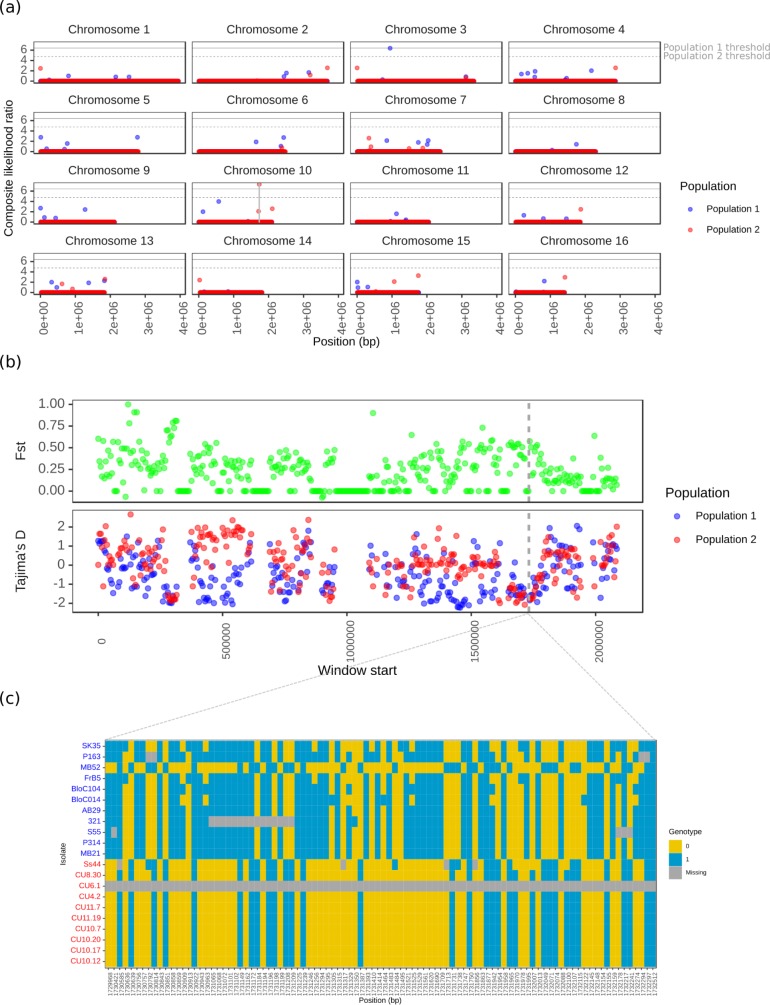
Selective sweep scan across the whole genome of *Sclerotinia sclerotiorum*. (a) Composite likelihood ratio (CLR, y axis) of a selective sweep occurring at all sites (x axis) across each of the 16 *Sclerotinia sclerotiorum* reference chromosomes. The blue points are for population 1 (North American and European isolates) and the red points are for population 2 (Australian isolates and a single Moroccan isolate). The horizontal grey lines represent the threshold CLRs from coalescent simulations using the best fit models of population demography. The solid grey line is for population 1 and the dashed grey line is for population 2. A single candidate hard selective sweep above the coalescent simulation threshold was identified on chromosome 10. It occurred in population 2 and is marked out with solid vertical grey lines. (b) Fst (green points) and Tajima’s D (blue and red points for population 1 and 2, respectively) statistics (y axis) for 50 Kb end to end windows (window start is on the x axis) across chromosome 10, where the single selective sweep was identified. The two vertical dashed grey lines represent the site of the selective sweep. Though Fst was not obviously higher in this region, Tajima’s D was lower than average in both populations. (c) Haplotype plot of the selective sweep region on chromosome 10. Rows are isolates, labelled to the left in blue for population 1 and red for population 2, and columns are biallelic SNP markers, with positions labelled on the x axis. Coloured blocks represent genotype values of 0 (reference, in yellow), 1 (alternative, in blue), or missing (grey). This shows a clear lack of genotypic diversity in population 2, which likely caused the lowered Tajima’s D and elevated CLR statistics at this site.

The CLR method first described by Nielsen et al. [4] was used to detect positive selective pressure in population 1 and population 2. The analysis was also run on 10,000 MS simulations to determine a threshold value for detection of positive selective pressure. The highest CLRs from the MS simulations were 6.41 for population 1 and 4.76 for population 2. In population 1, there were no observed CLRs that were above this threshold. However, in population 2, there was a single selective sweep that occurred on chromosome 10, with a CLR of 7.30 (Fig 5). This region spanned 5,035 bp of intergenic sequence (chromosome 10, 1,729,170 bp to 1,734,205 bp) and exhibited a particularly low Tajima’s D in both populations. However, F_st_ was not elevated in this region (Fig 5). The drop in Tajima’s D and likely reason for the higher CLR is observable in the haplotype frequency, plotted in Fig 5. Intriguingly, this region was missing from the isolate CU6.1, monomorphic in eight of the nine other Australian isolates, and similar in the Moroccan isolate Ss44. This contrasted the overall higher sequence diversity observed in population 1.

This single selective sweep was immediately downstream of a Major Facilitator Superfamily (MFS), sugar transporter-like domain (PF00083) (sscle_10g_079770, start: 1,734,268, stop: 1,736,172 (antisense strand)). Intriguingly, this selective sweep exhibited a potential transposable element insertion at 1,732,785 bp (1,483 bp downstream of sscle_10g_079770). We further investigated this insertion by visually inspecting read mappings surrounding the site and looking for discordant read pairs or gapped alignments. However, we found that the insertion was present in individuals in both populations. Therefore, we cannot make any strong conclusions about the nature of the selective sweep at this stage.

## Discussion

### Clustering of isolates by geographical origin

Spatial clustering of isolates has been previously demonstrated with genotypic markers such as multilocus haplotypes (MLHs). Carpenter et al. [59] showed that identical MLH fingerprints were shared only between isolates from the same farms in New Zealand. Carbone and Khon [60] suggested divergence between Norwegian populations of *S*. *sclerotiorum* present in two regions approximately 200 km apart. This was based on a lack of shared fingerprints between the regions, evolution of new fingerprints and increase in size of the intergenic spacer region over a two year period in a specific locale. Sun et al. [61] used random amplification of polymorphic DNA (RAPD) and unweighted pair-group mean analysis (UPGMA) to demonstrate that isolates from Anhui province in China formed a distinct cluster that was distant from another cluster formed from isolates from Poland and Canada. Carbone & Kohn [62] demonstrated divergence between isolates from Wild Buttercup in Norway and populations from different regions of the United States. Haplotypes present in these populations appeared to be exclusive to particular climates, suggesting environmental adaptation. In a further study, Malvárez et al. [63] showed divergence of a Californian population of *S*. *sclerotiorum* from the previously described North American populations. And, in a more recent study by Clarkson et al. [29], it was found that MLHs were not shared between isolates from the UK, Norway and Australia, possibly suggesting that these isolates belong to distinct populations.

In this study, we show that isolates from various geographically distant North American and European regions form a distinct genotypic cluster (referred to as population 1). The isolates from Australia, South Africa and Morocco formed a further distinct cluster with a few outliers. This cluster contained nine isolates from Australia and one from Morocco (referred to as population 2). This clustering may suggest adaptation of particular populations of *S*. *sclerotiorum* to different climates, as was suggested by Carbone et al. [62]. Although the various North American and European climates from which isolates in population 1 were sampled are quite different, they all exhibit a break in the season due to a cold or freezing winter and lack of an extensive period of drought (Köppen climate classifiers Cfb and Dfb). This contrasts the climates from which population 2 isolates were sampled, which are characterised by a break in season due to a hot summer with a significant dry period (Köppen climate classifiers Csa and Csb). Fragmentation of the ancestral population into these two populations may have occurred during the last glacial maximum.

The demographic modelling we performed suggested an ancient split between these two populations, perhaps more than 10 million years. The fact that the two populations are still derived from the same species is accounted for in the model by a moderate constant migration rate between populations, suggesting incomplete separation. The actual divergence time between these two populations cannot be currently determined, since there is no estimation of the mutation rate for *S*. *sclerotiorum* (which exhibits some evidence of the fungus-specific mutational process ‘Repeat Induced Point mutation’ [24]) and generation time is difficult to accurately infer. If the mutation rate in *S*. *sclerotiorum* is higher than that of yeast, the rate we used for model scaling, the divergence time of the two populations may be more recent and could coincide with the last glacial maximum. For example, applying a mutation rate 50-fold that of yeast leads to a divergence time on the order of tens of thousands of years; this is purely speculation at this stage. Overall, analyses would suggest that the two populations have been separated for a reasonable amount of time, most likely a long time prior to the appearance of the species in Australia with the spread of agriculture.

### Linkage disequilibrium decay indicates outcrossing in population clusters

*S*. *sclerotiorum* is homothallic, and therefore contains both mating types necessary for successful crossing. It is likely that this favours selfing of the fungus in natural populations, which would lead to extensive clonality. The extent to which outcrossing occurs in nature is unclear, but it has been demonstrated to occur under laboratory conditions. Carbone & Kohn [62] suggested that recombination in contemporary populations of *S*. *sclerotiorum* is infrequent. This is substantiated by numerous studies in which the same clones have been isolated in successive years, and others in which populations were shown to be composed predominantly of frequently occurring clones. Other studies have suggested that recombination may be more frequent in populations of *S*. *sclerotiorum*. For example, Attanayake et al. [64] suggested that linkage disequilibrium (LD) decay in seven genotypic groups of *S*. *sclerotiorum* from diverse geographical regions may be evidence of extensive outcrossing.

In the current study, LD decay in two genotypic clusters suggested some outcrossing between clonal lineages. In both populations, LD decayed to halfway between its maximum and minimum at approximately 20 Kb. Furthermore, recombination rates for the two populations were estimated to be 0.000172 and 0.0000123. These rates are lower than those observed for *Microbotryum* spp. (from 0.00102 to 0.0116) but support the observation of linkage disequilibrium decay with physical distance.

### A single selective sweep in population 2 may be evidence of selection pressure on a Major Facilitator Superfamily transporter

In both populations there was some evidence of outcrossing and meiotic exchange of alleles. Although the samples were likely not drawn from completely panmictic populations, they at least partially met the assumptions of selective sweep scans due to the observed LD decay and recombination rates. To conduct selective sweep scans, we used the method developed by Nielsen et al. [4], which is reasonably robust against assumptions of both underlying demography and recombination rate. We also fit a demographic model to the allele frequency data to further control for false signatures of selection.

Overall, there was scant evidence of hard selective sweeps in the *S*. *sclerotiorum* genome. Only a single point exhibited a CLR above the threshold in population 2. Furthermore, this point, with a CLR of 7.3, barely met the threshold of 4.76. This contrasts the abundant selective sweeps observed in *Z*. *tritici*–in one population from Israel, 126 selective sweeps were found in this species. Additionally, the thresholds used in this study were percentile based rather than simulation based, and were generally far higher than the thresholds we used for *S*. *sclerotiorum*. For example, in the Australian population of *Z*.*tritici* analysed, the threshold CLR was 389.

The question then is why were strong selective sweeps so numerous in *Z*. *tritici* and distinctly absent from *S*. *sclerotiorum*? This may be a result of several features of the life cycles and reproductive modes of these species. Firstly, *S*. *sclerotiorum* undergoes a single sporulation event per year, releasing a cloud of ascospores from apothecia. This contrasts the multiple sporulation events that can occur in *Z*. *tritici* via splash-dispersed pycnidiospores. The single sporulation event in *S*. *sclerotiorum* may lead to a smaller amount of mutant progeny in a single season, and thus reduce the adaptive capacity of this species. This could be the result of an adaptation to broad host range pathogenesis in *S*. *sclerotiorum*, which would favour lineages with increased overall fitness on multiple hosts over lineages that are adapted to a single host that is only transiently present temporally [25].

Secondly, *Z*. *tritici* undergoes multiple sexual crosses per season, whereas *S*. *sclerotiorum* likely undergoes a single, essentially clonal reproductive event in many seasons. The sexual reproduction in *Z*. *triciti*, coupled with its multiple sporulation events would lead to a classic hard selective sweep scenario under which favourable mutations arise and are rapidly swept through populations, losing alleles either side through recombination. In a species where reproduction often occurs clonally, even if selective pressure were particularly pronounced for a single allele, hard selective sweeps may be less easy to observe. This is because of two main reasons: spread of a favourable allele into new haplotypes would be less likely, and LD would extend far beyond the swept region making it difficult to detect. Furthermore, a lack of spread to new haplotypes would reduce the likelihood of loss of new deleterious alleles linked with the advantageous allele, leading to a reduction in fitness and halt of the sweep. Similarly, different haplotypes with the same selective advantage as the allele being swept might arise in alternate clonal lineages which, being also selected for, would reduce the hard selective sweep signature in a population.

The single selective sweep that was detected was within 10 Kb of only a single gene, a MFS transporter (possibly of sugars). Intriguingly, several MFS transporters were associated with selective sweeps in both *Z*. *tritici* and *Microbotryum* spp. This is particularly interesting as many studies have implicated fungal MFS transporters in resistance to multiple fungicides [65,66]. Since there is no known resistance of *S*. *sclerotiorum* to fungicides in Australia, it is difficult to speculate about the significance of this finding. Although it is possible for resistance to develop towards chemistries not registered for a specific pathogen but sprayed for another on the same crop.

Alternatively, the MFS transporter could be involved in interaction with host plants. For example, in *Colletotrichum lindemuthianum*, an MFS sugar transporter was shown to be important for acquiring nutrients in the necrotrophic phase of infection [67]. It is possible that changes in regulation of the *S*. *sclerotiorum* MFS were selected for as it allows utilisation of sugars produced by a host species or cultivar planted more commonly in Australia. At best, all we can do currently is speculate.

In conclusion, we found evidence of a likely ancient population split between *S*. *sclerotiorum* isolates from Australia and Morocco and Europe and North America. The two populations exhibited little evidence of hard selective sweeps, except a possible candidate near an MFS transporter in the Australian and Moroccan group. We speculate that this allele may be important for nutrient uptake from the host.

## Supporting information

S1 File **Fig A. Read depths across variants called in the *Sclerotinia sclerotiorum* genome.** Blue vertical lines represent a depth of 150 x, which was the maximum for retaining a variant. We chose this value as it was approximately placed at the upper end of the distribution of the majority of variants called across all populations. **Fig B. Phylogenetic tree placing isolates used in this study in a clade with *Sclerotinia sclerotiorum*.** The node labels represent bootstrap support from 1000 bootstraps.
(PDF)

S1 Table Information on isolates used in this study. (XLSX)

S2 Table Accessions of *HSP60* sequences from fungi used for construction of the phylogenetic tree in Fig A in S1 File. (XLSX)

S3 Table Recombination rates determined for the two *Sclerotinia sclerotiorum* populations using LDHat. (XLSX)

S4 Table Parameter estimates and log likelihoods of the demographic models fit to the *Sclerotinia sclerotiorum* SNP data. (XLSX)
