# Supplementary material for: A whole genome scan of SNP data suggests a lack of abundant hard selective sweeps in the genome of the broad host range plant pathogenic fungus Sclerotinia sclerotiorum
Source: PLoS One. 2019 Mar 28;14(3):e0214201. doi: 10.1371/journal.pone.0214201 (PMC6438532; doi:10.1371/journal.pone.0214201)
Supplement: S1 File — Fig A. Read depths across variants called in the Sclerotinia sclerotiorum genome. Blue vertical lines represent a depth of 150 x, which was the maximum for retaining a variant. We chose this value as it was approximately placed at the upper end of the distribution of the majority of variants called across all populations. Fig B. Phylogenetic tree placing isolates used in this study in a clade with Sclerotinia sclerotiorum. The node labels represent bootstrap support from 1000 bootstraps. (PDF) [file pone.0214201.s001.pdf]

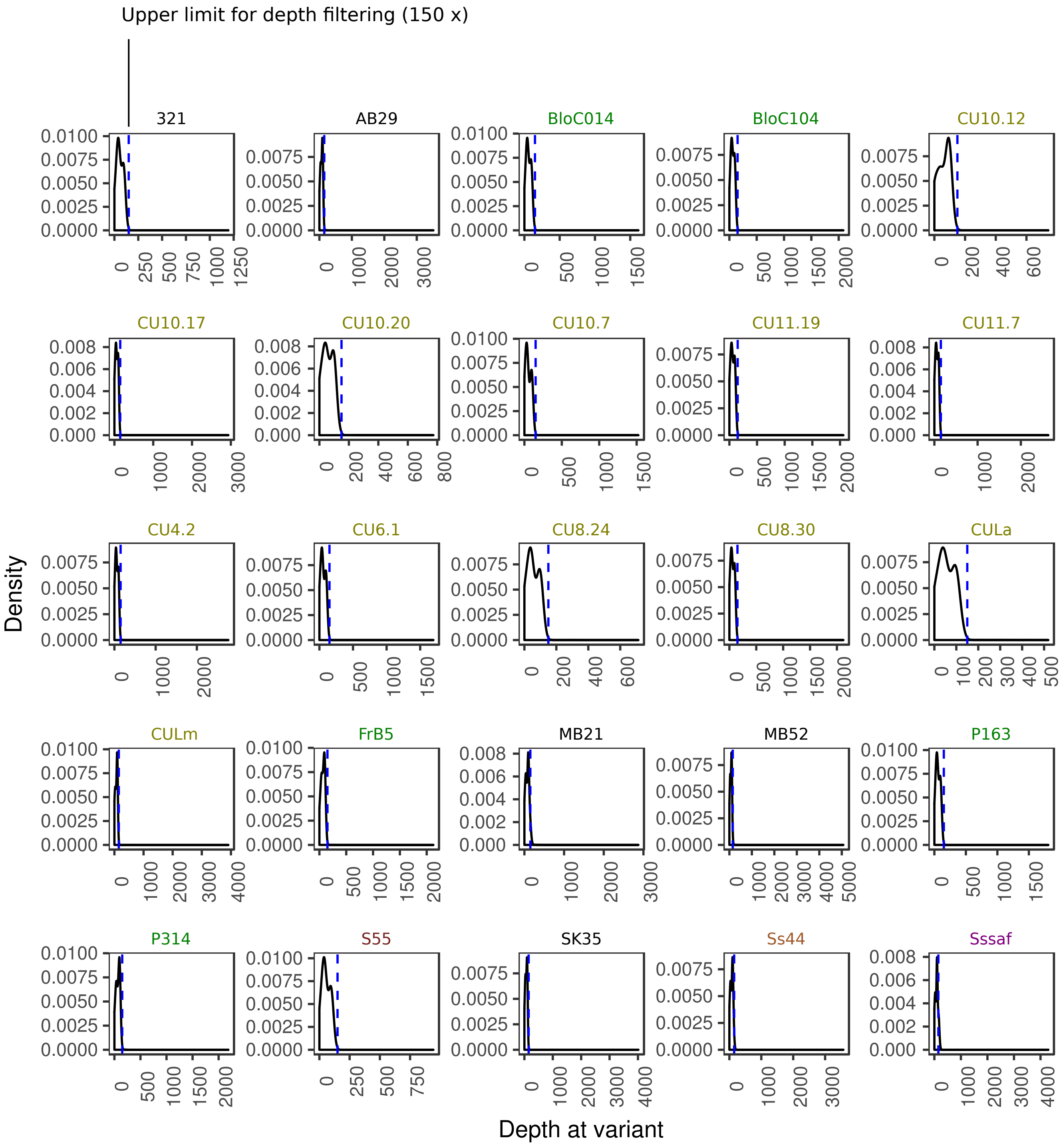
Upper limit for depth filtering (150 x)
321
AB29
BloC014
BloC104
CU10.12
CU10.17
CU10.20
CU10.7
CU11.19
CU11.7
CU4.2
CU6.1
CU8.24
CU8.30
CULa
CULm
FrB5
MB21
MB52
P163
P314
S55
SK35
Ss44
Sssaf
Density
Depth at variant

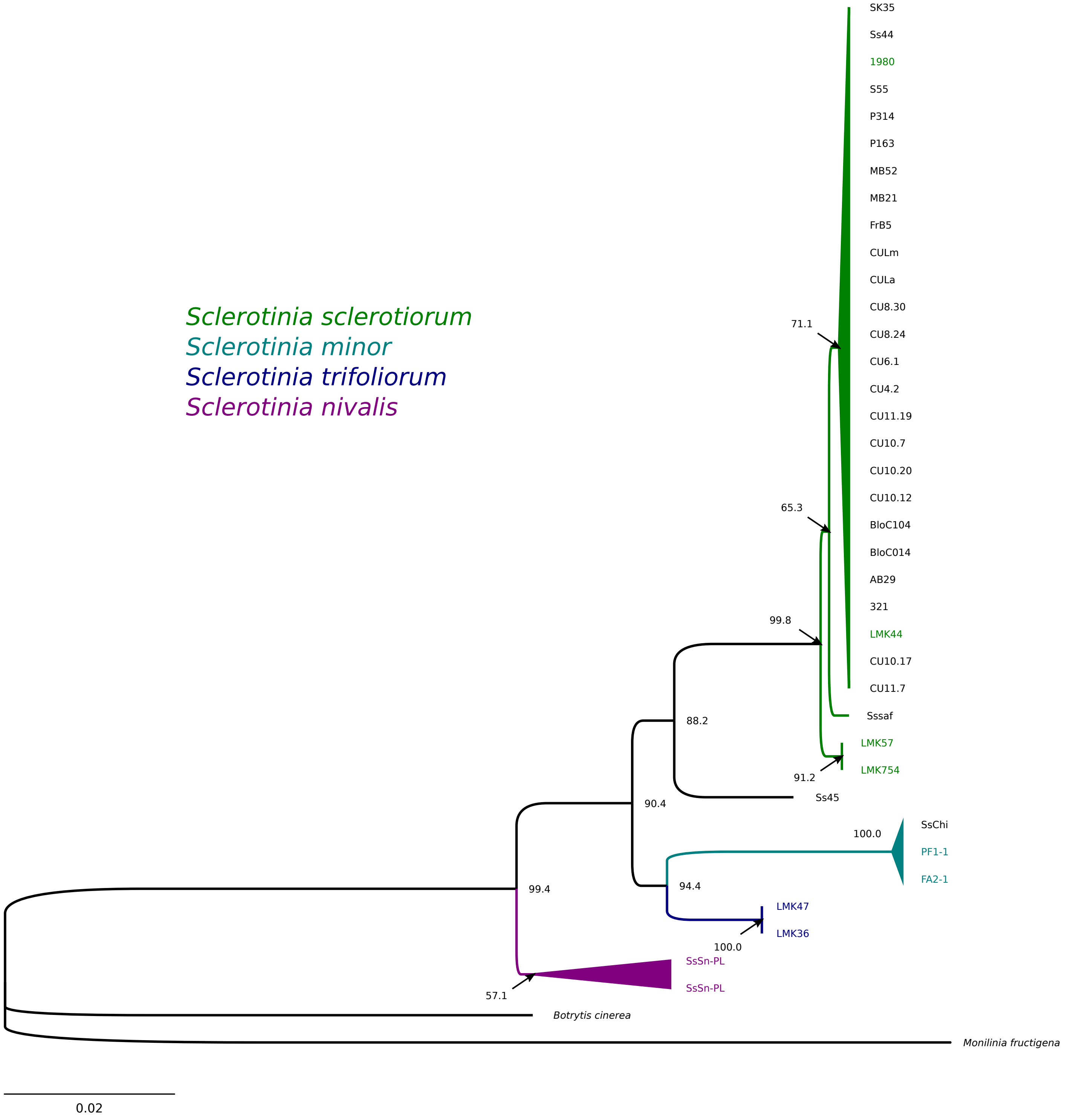
Sclerotinia sclerotiorum
Sclerotinia minor
Sclerotinia trifoliorum
Sclerotinia nivalis
SK35
Ss44
1980
S55
P314
P163
MB52
MB21
FrB5
CULm
CULa
CU8.30
CU8.24
CU6.1
CU4.2
CU11.19
CU10.7
CU10.20
CU10.12
BloC104
BloC014
AB29
321
LMK44
CU10.17
CU11.7
Sssaf
LMK57
LMK754
Ss45
SsChi
PF1-1
FA2-1
LMK47
LMK36
SsSn-PL
SsSn-PL
Botrytis cinerea
Monilinia fructigena
71.1
65.3
99.8
88.2
91.2
90.4
100.0
94.4
100.0
99.4
57.1
0.02
